# Task-induced brain activity in aphasic stroke patients: what is driving recovery?

**DOI:** 10.1093/brain/awu163

**Published:** 2014-06-28

**Authors:** Fatemeh Geranmayeh, Sonia L. E. Brownsett, Richard J. S. Wise

**Affiliations:** Computational Cognitive and Clinical Neuroimaging Laboratory, Division of Brain Sciences, Imperial College London, Hammersmith Hospital, London, W12 0NN, UK

**Keywords:** aphasia, functional brain mapping, functional recovery, cognitive control, attention

## Abstract

The estimated prevalence of aphasia in the UK and the USA is 250 000 and 1 000 000, respectively. The commonest aetiology is stroke. The impairment may improve with behavioural therapy, and trials using cortical stimulation or pharmacotherapy are undergoing proof-of-principle investigation, but with mixed results. Aphasia is a heterogeneous syndrome, and the simple classifications according to the Broca-Wernicke-Lichtheim model inadequately describe the diverse communication difficulties with which patients may present. Greater knowledge of how intact neural networks promote recovery after aphasic stroke, either spontaneously or in response to interventions, will result in clearer hypotheses about how to improve the treatment of aphasia. Twenty-five years ago, a pioneering study on healthy participants heralded the introduction of functional neuroimaging to the study of mechanisms of recovery from aphasia. Over the ensuing decades, such studies have been interpreted as supporting one of three hypotheses, which are not mutually exclusive. The first two predate the introduction of functional neuroimaging: that recovery is the consequence of the reconstitution of domain-specific language systems in tissue around the lesion (the ‘perilesional’ hypothesis), or by homotopic cortex in the contralateral hemisphere (the ‘laterality-shift’ hypothesis). The third is that loss of transcallosal inhibition to contralateral homotopic cortex hinders recovery (the ‘disinhibition’ hypothesis). These different hypotheses at times give conflicting views about rehabilitative intervention; for example, should one attempt to activate or inhibit a contralateral homotopic region with cortical stimulation techniques to promote recovery? This review proposes that although the functional imaging data are statistically valid in most cases, their interpretation has often favoured one explanation while ignoring plausible alternatives. In our view, this is particularly evident when recovery is attributed to activity in ‘language networks’ occupying sites not observed in healthy participants. In this review we will argue that much of the distribution of what has often been interpreted as language-specific activity, particularly in midline and contralateral cortical regions, is an upregulation of activity in intact domain-general systems for cognitive control and attention, responding in a task-dependent manner to the increased ‘effort’ when damaged downstream domain-specific language networks are impaired. We further propose that it is an inability fully to activate these systems that may result in sub optimal recovery in some patients. Interpretation of the data in terms of activity in domain-general networks affords insights into novel approaches to rehabilitation.

## Abstract (precis)

Based on the interpretation and reinterpretation of published functional neuroimaging and clinical neuropsychological data, Geranmayeh *et al*. argue that recovery from aphasic stroke may be due as much to the function of high-order, domain-general networks as to recovery of language-specific networks. This distinction has implications for rehabilitation.

## Introduction

It is claimed that one-third of all stroke patients have an aphasic deficit as part of their presenting symptoms (Laska *et al.*, 2001; Pedersen *et al.*, 2004; Lazar *et al.*, 2008). Although half of these patients recover much or all of their language function, the remainder are left with a persisting and disabling impairment of communication. It is estimated that in the USA and the UK alone, the current prevalence of post-stroke aphasia is 1 million and 250 000, respectively.

Not surprisingly, most studies and subsequent meta-analyses that have investigated prognosis have concluded that the initial severity of the overall stroke deficit, and of the aphasic deficit specifically, and the size of the lesion afford the most reliable indicators of prognosis (Pedersen *et al.*, 1995; Lazar *et al.*, 2008; Maas *et al.*, 2012; Plowman *et al.*, 2012). However, these factors explain only about one-third of the variability in recovery from aphasia, with lesion volume contributing little to the variance in one multiple regression analysis on 22 patients (Lazar *et al.*, 2008). The contribution of demographic factors, such as sex, age, premorbid intelligence and handedness, as explanatory variables for prognosis appears to contribute even less to the prediction of final outcome (Plowman *et al.*, 2012); although in one large series it has been demonstrated that increasing age is associated with a worse prognosis (Knoflach *et al.*, 2012). Periodically it is reported that infarction of certain small regions reliably result in a specific and lasting aphasic deficit, but such reports may attract further studies suggesting that the anatomical-behavioural association is not as strong as originally suggested (for example, see Dronkers, 1996, and the subsequent paper by Hillis *et al.*, 2004). Publication bias also adds to the uncertainty about ‘critical’ lesion sites. For example, infarction of a few cubic millimetres of left ventral occipito-temporal cortex and its connections to left and right visual cortex have become associated with a particularly severe and persistent impairment of reading (Binder and Mohr, 1992); but there is no way of ascertaining whether other cases with lesions of the same region were not reported because their alexia was mild or transient. Patients are selected on the basis of a theoretically interesting deficit, not on an absence of a deficit.

If there is uncertainty about the value of routine clinical and imaging measures in predicting recovery from aphasia, there is even greater uncertainty about the value of therapeutic intervention. Behavioural therapy receives support in the latest Cochrane library review on the topic (Brady *et al.*, 2012). Although there is an emphasis on more rather than fewer hours of practice (Bhogal *et al.*, 2003), compliance with prolonged (and often tedious) exercises outside an intensive research setting may be difficult to achieve. There is some enthusiasm for either drug therapy (Berthier *et al.*, 2011) or cortical stimulation techniques, using transcranial magnetic or direct current stimulation (Hamilton *et al.*, 2011; Elsner *et al.*, 2013), but the studies to date are too small to allow any confident conclusions to be drawn from the results. There is also the concern that completed studies that demonstrate a null result, remain unpublished.

Uncertainties regarding prognosis and the efficacy of therapeutic interventions over and above ‘natural’ recovery would be reduced if the mechanisms of recovery were better understood. This review will consider the role of functional neuroimaging in fulfilling this goal. One strong conclusion that we will draw is that interpretation of the data has often been too narrow. In particular, we build on prior evidence from parallel studies in the field of cognitive neuroscience that have often been overlooked in the field of aphasiology, drawing attention to the contribution of domain-general systems acting on damaged domain-specific language networks. In essence this is a refinement of a common bedside clinical intuition; that if executive function and attention are impaired in an aphasic patient, due to the lesion distribution or co-existing age-related cognitive decline, microangiopathic cerebrovascular disease or neurodegenerative disease, the worse the prognosis and the response to rehabilitative interventions.

## The introduction of functional neuroimaging to language research

The modern era of functional neuroimaging was heralded by a publication in *Nature* (Petersen *et al.*, 1988)*,* now cited ∼2000 times. This reported a study on healthy participants using PET and what became the classic ‘subtraction’ design, whereby activity in one behavioural state is subtracted from another to identify the anatomical location of the cognitive function of interest. Two semantic tasks were reported as showing a conjunction of activity in the left inferior frontal gyrus (IFG). The authors concluded that the left IFG, largely incorporating ‘classic’ Broca’s area [Brodmann areas (BA) 44 and 45] and adjacent ventrolateral inferior frontal cortex, ‘participates in processing for semantic association’. In the discussion of this result, the authors explicitly contrasted their result with the neurological model of single word processing proposed by Geschwind (1965*a*, *b*), who argued that the processing of semantic associations is located at the other extreme of the left sylvian fissure, in inferior parietal cortex.

The paper of Petersen and colleagues (1988), and a further publication by the same group (Petersen *et al.*, 1990), were very influential in terms of introducing a new methodology to human neuroscience and advancing our understanding of the functional anatomy of language. However, most would now agree that one plausible interpretation of their studies, that representations of semantic knowledge are stored in the left IFG, is an unlikely explanation for their results. Semantic processing is likely to be very distributed, with anterior and ventral temporal cortex playing a key role (McClelland and Rogers, 2003; Acosta-Cabronero *et al.*, 2011; Lambon Ralph *et al.*, 2012). What has become evident is that the left IFG is a core component in processes involved in accessing these representations in a context-dependent manner. Therefore, alternative proposals were made based on further experiments, using the alternative (and now almost universal) methodology of functional MRI: for example, that it is not the retrieval of semantic knowledge *per se* that results in increased left IFG activity, but rather selection of information amongst competing alternatives from semantic memory (Thompson-Schill *et al.*, 1997); or, alternatively, that the left IFG controls semantic retrieval irrespective of whether the selection is from amongst competing representations (Wagner *et al.*, 2001). As another example, an interpretation based on retrieval processes rather than on representations was also made in relation to the effects of semantic ambiguity in sentence comprehension. Thus, ‘the shell was fired towards the tank’ is effortlessly accepted as meaningful, despite the two nouns and the verb having a number of alternative meanings. Were a listener to select incorrectly from among these alternative meanings, the sentence would be perceived as nonsensical. When participants in a functional MRI study parsed ‘high-ambiguity’ sentences, they demonstrated increased left IFG activity compared to when they heard ‘low-ambiguity’ sentences, comprised of words with only one meaning that were otherwise matched for linguistic variables, such as word frequency and imageability (Rodd, 2005).

**Figure 1:**
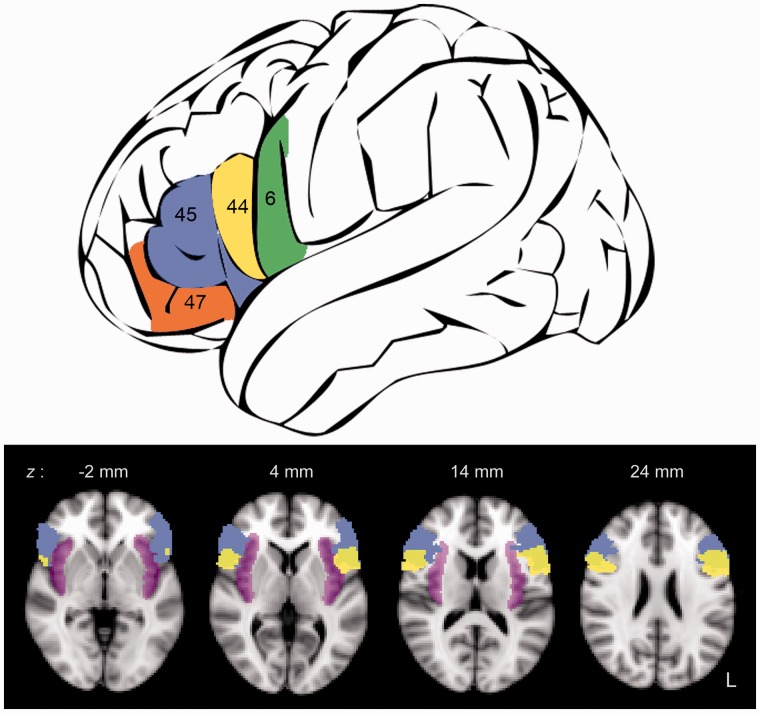
Broca’s area, adjacent frontal operculum and the insula are commonly activated in neuroimaging studies employing language tasks. Activations in these regions are often interpreted as activity in a larger Broca’s area. *Top* panel shows a schematic drawing of the lateral view of the left hemisphere and the position of the classic Broca’s area defined as encompassing Brodmann’s areas (BA) 44 (yellow) and 45 (blue) and adjacent cortex in BA 47 (orange) and ventral BA 6 (green). *Bottom* panel shows axial slices from T_1_-weighted MRI images in Montreal Neurological Institute standard space, superimposed with bilateral BA 44 (yellow) and 45 (blue) from the Juelich histological atlas (http://www.fmrib.ox.ac.uk/fsl/) and insular cortices (magenta) from the Harvard-Oxford cortical structural atlas (http://www.fmrib.ox.ac.uk/fsl/). The probabilistic maps of these brain regions overlap considerably. Numbers attached to the axial slices represent the coordinates in the *z*-plane above the anterior-posterior commissural line.

Interpretations of the function of Broca’s area have continued as a major preoccupation of functional neuroimaging research [entering the search terms ‘Broca’s AND (fMRI OR PET)’ in PubMed returns 731 references]. Given its pre-eminence as a cortical ‘module’ for language, most functional neuroimaging research directed at this region has been by scientists who are preoccupied with the processing of phonology, words or syntax, and how separable subcomponents of Broca’s area are involved in these different linguistic processes (Vigneau *et al.*, 2006). Nevertheless, activity in Broca’s area has also come to feature prominently in studies that have hypotheses and interpretations unrelated to language-specific processing. Broca’s area is anatomically and neurochemically heterogeneous (Amunts *et al.*, 1999; Amunts and Zilles, 2012). Further, the anatomical and functional connectivity of this region is widely distributed (Lemaire *et al.*, 2013), particularly if one views Broca’s area as including not only the pars opercularis and triangularis, but also the adjacent lateral orbital, ventral premotor and anterior insular cortices, various combinations of which appear in publications referring to Broca’s area (Fig. 1). It is, therefore, not surprising that this region activates in response to non-linguistic as well as linguistic stimuli. Thus, for example, this region figures prominently in a functional MRI study that investigated the processing of the hierarchical organization of behaviour (Koechlin and Jubault, 2006). Humans have the capacity to combine sequences of subordinate stimuli into increasingly complex superordinate structures, which in turn influence the behaviour of the observer. This occurs across multiple domains (for example, playing chess or listening to a passage of music); and, of course, also during the processing of language, when a sequence from a limited repertoire of phonemes can be used by the speaker to elicit emotions and actions in the listener. Koechlin and Jubault’s (2006) study demonstrated that both the left and right IFG were implicated in the ‘chunking’ of, and responding in a rule-based manner to, sequences of visual stimuli, with a posterior-to-anterior gradient for simpler and more complex sequences. The bilateral response in this study is of interest, as the comprehension and production of language are typically only associated with increased activity in the left IFG (Hickok and Poeppel, 2007), suggesting that hierarchical sequencing of the elements of language carried out in the IFG (otherwise known as ‘unification’, see Hagoort, 2005) has become the property of a domain-specific and lateralized subsystem embedded within a more bilateral domain-general network.

Broca’s area, through its anatomical and functional connectivity, may be involved in a number of processes engaged in the comprehension and production of single words and sentences. However its functional heterogeneity has been demonstrated in a further functional MRI study on healthy participants. This set out to determine the response of the left pars opercularis and triangularis across a wide range of language and non-language tasks. Within these two regions, although subcomponents were active only during a language task, others were active across all tasks (Fedorenko *et al.*, 2012).

**Figure 2:**
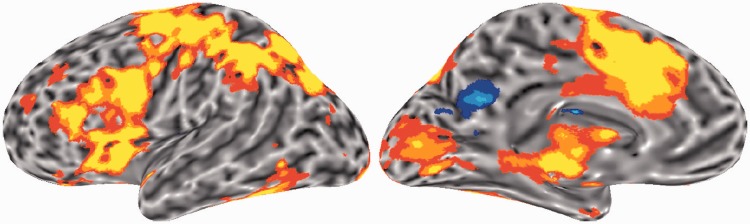
Regions showing a positive correlation between reaction times and activity during a lexical decision task from the study of Binder *et al.* (2005) © 2005 by the Massachusetts Institute of Technology. Left hemisphere lateral and medial views are shown; the distribution of activity throughout the right hemisphere was very similar. Red–yellow colours indicate positive correlations, blue colours indicate negative correlations. Regions that show a positive correlation include bilateral anterior insular cortices and the adjacent inferior frontal gyrus, the middle frontal gyri, extending posteriorly to the precentral sulci and the intraparietal sulci, dorsal anterior cingulate cortex and adjacent superior frontal gyrus. These regions are now considered to be components of the domain-general networks, they are known as cingulo-opercular and fronto-parietal control networks, and it is proposed that these networks are responsible for processes associated with domain-general cognitive control and attention.

The additional observation in the study of Fedorenko and colleagues (2012) was that the harder a task, the greater the activity in Broca’s area. Often, activity associated with task difficulty, as reflected in the reaction times and/or error rates, has been considered a confound when the motivation for a study is to determine the functional anatomy of domain-specific processes. Dealing with this confound can be achieved, at least in part, in several ways: ensuring that in a subtraction design the activation and baseline tasks are matched for reaction times and error rates; failing that, by regressing out these variables in the analysis; or, if the study was an event-related functional MRI study, simply by discarding the functional images from the trials with prolonged reaction times or error rates. Occasionally, researchers have included the activity associated with task difficulty (sometimes known as the ‘time-on-task’ effect) alongside their main findings. One example of such a publication, and of interest in terms of the rest of this review, is that by Binder and colleagues (2005). The study investigated the functional anatomy of domain-specific representations for concepts conveyed by abstract compared to concrete words. The healthy participants performed a lexical decision task on concrete, abstract words and non-words (the last being the baseline condition). The length of the reaction times for making the decision were: non-words > abstract words > concrete words. In the main analyses, the reaction times were included in the regression model to account for any effect of time-on-task in the main result. However, an analysis investigating the effect of reaction times across all trials was also included. This analysis demonstrated positive correlation between reaction times and distributed activity in regions including bilateral anterior insular cortices and the adjacent IFG (anterior insular/IFG), the middle frontal gyri, extending posteriorly to the precentral sulci and the intraparietal sulci, dorsal anterior cingulate cortex (ACC) and adjacent superior frontal gyrus (dorsal ACC/SFG) (Fig. 2). These regions form two networks, the cingulo-opercular and one of several fronto-parietal networks, that have now been studied in some detail by groups interested in domain-general attention and cognitive control of domain-specific processes (Dosenbach *et al.*, 2007, 2008; Menon and Uddin, 2010; Duncan, 2013). We will now summarize this literature, the relationship of these networks to another system known as the default mode network, and end the next section by discussing two recent studies on healthy participants that specifically related activations during language functional MRI tasks to these networks.

## Domain-general cognitive control brain systems

The human brain contains cortical regions that are specialized for domain-specific processes: for example, early visual or auditory processing, orthographical perception or motion perception. However, over the past decade there has been considerable research on domain-general brain systems that are engaged across a wide range of cognitive tasks.

The existence of domain-general cortical regions is reasonably well established. One influential hypothesis is that a set of these regions, termed the ‘Multiple-Demand’ system, rapidly adapt to exert top–down control during a broad range of tasks (Duncan and Owen, 2000; Duncan, 2010, 2013; Fedorenko *et al.*, 2013). The Multiple-Demand system relates to general psychological constructs like intelligence quotient, cognitive flexibility, behavioural inhibition, and attentional control (Duncan, 2005; Hampshire *et al.*, 2012). This system is minimally engaged when performing an overlearned (habitual) task, but comes into play when solving novel problems, when task conditions change and habitual responses require modification, and more generally, whenever tasks require a greater level of top–down control. One can conceptualize that language tasks may engage all of these processes, particularly when domain-specific resources alone do not suffice, either in healthy participants performing difficult metalinguistic tasks (Hampshire *et al.*, 2013) or when the habitual functioning of language networks have been impaired by pathology.

**Figure 3:**
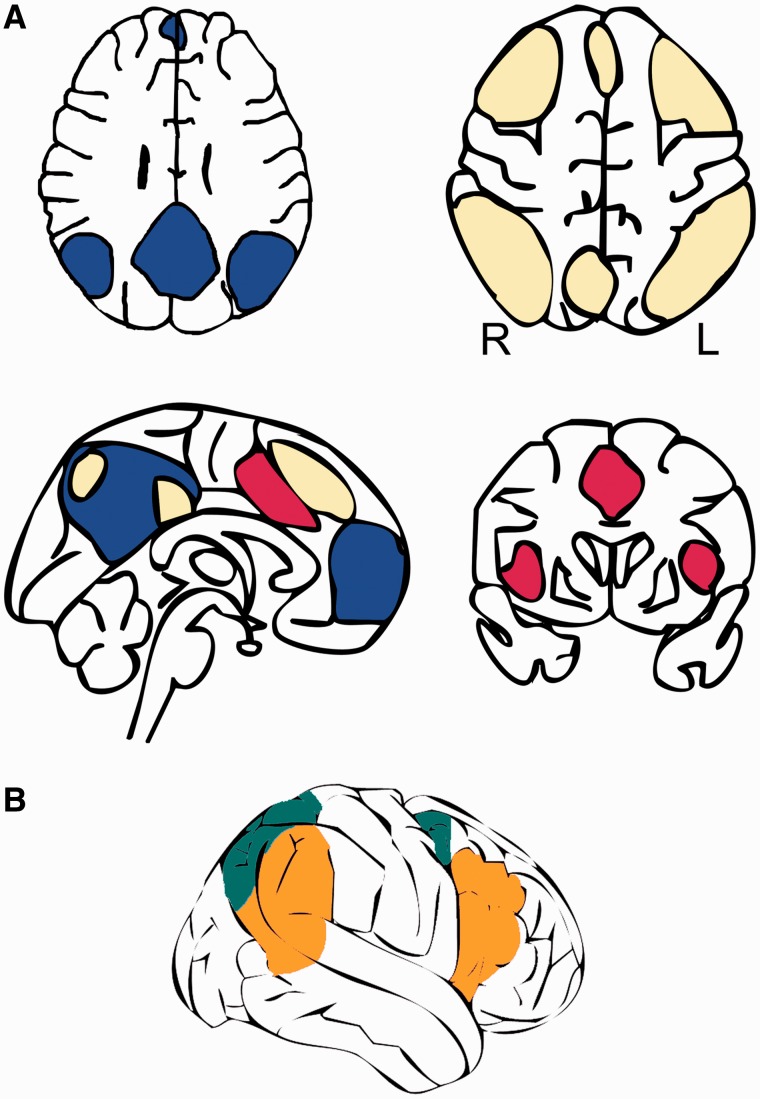
Schematic drawing of the typical spatial distribution of domain-general networks that may be engaged during neuroimaging of language tasks in healthy controls as well as aphasic patients. Many functional neuroimaging studies depict these networks as spatially overlapping. (**A**) The coloured networks are the Default Mode Network in blue, the fronto-parietal control network in yellow, and the cingulo-opercular network in red. The Default Mode Network is a ‘task-negative’ network that is deactivated during task performance on stimuli. Although they are functionally separable networks, the fronto-parietal control and cingulo-opercular networks often co-activate (see Fig. 2), and are considered to exert attention and executive control, and other processes involved in making a decision, selecting a response, and monitoring and correcting for errors. (**B**) Attentional networks can be divided into two broad systems; the dorsal attention network, in green, is thought to be a goal-driven ‘top–down’ attentional system, and is distributed symmetrically between the two hemispheres. The ventral attention network, in orange, is considered a stimulus-driven or ‘bottom–up’ attentional system, and largely lateralized to the right hemisphere.

The regions in the Multiple-Demand system include bilateral intraparietal sulcus, inferior frontal sulcus, anterior insula and adjacent frontal operculum, and the presupplementary motor area and adjacent dorsal anterior cingulate (SMA/dorsal ACC). The regions in the Multiple-Demand cortex have been further fractionated by some authors into subcomponents (Hampshire and Owen, 2006; Hampshire *et al.*, 2012). Depending on the exact task demands, these regions activate to varying degrees; however, the same subregions commonly co-vary in activation levels across tasks (Hampshire *et al.*, 2012) and even across time during rest (Dosenbach *et al.*, 2007). These intrinsic networks have been variously named as ‘task positive’ (Fox, 2005), ‘task-activation ensemble’ (Seeley *et al.*, 2007), or a ‘task control’ network (Dosenbach *et al.*, 2007). These are sometimes further divided into subnetworks including ‘fronto-parietal control,’ ‘dorsal attention’, and ‘cingulo-opercular’ (Dosenbach *et al.*, 2007; Vincent *et al.*, 2008; Power *et al.*, 2011; Power and Petersen, 2013). The exact mental processes that are mediated by these domain-general cortical and subcortical systems have not yet been clearly defined (Hampshire *et al.*, 2012). Similarly the use of over-specified labels may fail to capture the broader contributions of these networks to domain-general control. We will now briefly describe these networks (also see Fig. 3).

A combination of co-activation of cortical regions that overlap with the Multiple-Demand system is termed by some as the ‘fronto-parietal control’ (Dosenbach *et al.*, 2007, 2008; Power *et al.*, 2011; Power and Petersen, 2013) or ‘executive control’ network (Seeley *et al.*, 2007). This network incorporates left and right anterior and dorsolateral prefrontal cortices and the intraparietal sulci/adjacent dorsal inferior parietal cortices. The activity in the fronto-parietal control system has been attributed to initiation of task performance, and adjustment of cognitive control on a continuing trial-by-trial basis.

The ‘dorsal attention network’ (DAN), includes occipito-temporal cortex, the superior parietal lobule, intraparietal sulcus and frontal eye field in each hemisphere. Activity in this region has been mainly studied using goal-directed top–down selection of visual stimuli based on internal goals and shifts of spatial attention (Corbetta and Shulman, 2002; Corbetta *et al.*, 2008; Vincent *et al.*, 2008). This is in contrast to a predominantly right-lateralized ‘ventral attention network’ that also incorporates the junction of the inferior parietal lobe with posterior temporal cortex. This system is engaged in stimulus driven attention and detects salient and behaviourally relevant stimuli (Corbetta and Shulman, 2002; Corbetta *et al.*, 2008; Singh-Curry and Husain, 2009). These two networks overlap spatially with a set of regions implicated in processes that require attention sustained over time. Regions in both these networks have been proposed to work together during tasks that depend on vigilant attention (Langner and Eickhoff, 2013). However, the exact processes that they support remain the topic of much debate.

The co-activation of a network of brain regions in the dorsal ACC and adjacent medial superior frontal gyrus (dorsal ACC/SFG) and bilateral anterior insular and adjacent inferior frontal gyrus has been termed the cingulo-opercular network (Dosenbach *et al.*, 2007, 2008; Power *et al.*, 2011; Power and Petersen, 2013). The co-activation of a similar set of regions have been termed the ‘salience system’ (Seeley *et al.*, 2007; Menon and Uddin, 2010). These regions can co-activate with the regions in the fronto-parietal control network (Vincent *et al.*, 2008). The activity in this network has been attributed to goal-directed behaviour through the stable maintenance of task sets, or detection of salient events. In this review we adopt the label, cingulo-opercular network that focuses on anatomical description rather than the more controversial process-based labels (e.g. salience network).

We will now focus in more detail in one region that has been observed to be active in many language studies, and that is part of the domain-general cingulo-opercular network, namely the dorsal ACC/SFG (Braun *et al.*, 1997; Warburton *et al.*, 1999; Blank *et al.*, 2002; Geranmayeh *et al.*, 2012; Price, 2012). In addition to language tasks, this region is activated by many non-linguistic cognitive tasks, such as processing of emotion, pain, attention, motor control, memory, reward, and the monitoring of responses and errors (Paus, 2001; Rushworth *et al.*, 2003; Ridderinkhof, 2004; Kennerley *et al.*, 2006; Alexander and Brown, 2011; Torta and Cauda, 2011; Løvstad *et al.*, 2012). It remains unclear the amount to which the responses to these very disparate stimuli are processed by a common systems or by anatomically overlapping but functionally distinct subsystems within the dorsal ACC/SFG (Torta and Cauda, 2011). However, a small number of functional MRI studies on healthy participants have study designs that allowed the investigators to explicitly dissociate language-specific functional MRI activity from that related to domain-general processes involving the cingulo-opercular network. These studies and their results are summarized in Table 1.

**Table 1:** Functional MRI studies on healthy participants that explicitly dissociated language-specific functional MRI activity from that related to non-linguistic or domain-general processes

	Task [number of levels of task difficulty]	Outcome	Reported MNI coordinates of activity in regions associated with the cingulo-opercular network (*x*,*y*,*z*)		
			ACC	Right insula	Left insula
Eckert *et al.* (2009)	Visual attention: Eriksen Flanker task [two]	The same frontal regions (ACC, R aI/IFG, R dlPFC) were engaged, regardless of task.	Visual task −3,45,18	Visual task 39,3,−3	–
	Single word recognition: repeating heard filtered words [four]	Activity in these regions increased with task difficulty.	Language task 9,27,30	Language task 30,21,9	–
Erb *et al.* (2013)	Repeating heard sentences [two: vocoded versus clear sentences]	The difficult verbal task was associated with activity in the cingulo-opercular network. Parts of this network (dorsal ACC/SFG and left aI/IFG) also showed increased activity as the non-verbal task became more difficult. This activity was attributed to domain-general cognitive control.	−6,17,49	33,23,1	−30,23,−2
	Non-speech sound amplitude discrimination using button responses [seven]				
Baumgaertner and Hartwigsen (2013)	Three tasks (stimuli were presented in two domains: written and heard words):		−3,33,39	36,21,−6	−42,21,−6
	Perceptual manipulation judge ment: detect pitch or font size change	Right pars opercularis responded to non-linguistic perceptual processing.			
	Semantic judgement	There was greater activity in regions in a cingulo-opercular network for the perceptually manipulated stimuli during the perceptual processing but not phonological or semantic processing.			
	Phonological judgement	Activity in the left and right anterior insulae, was present for all three decision tasks on all stimuli relative to ‘rest’.			
Vaden *et al.* (2013)	Repeating words that were heard over background babble [two]	Activity in the cingulo-opercular network increased in proportion to percentage errors and task difficulty.	−7,31,40	42,27,−10	−42,24,−4
		Elevated cingulo-opercular activity increases the likelihood of immediate correct word recognition.	−1,35,34	43,21,−9	–
Piai *et al.* (2013)	Vocal picture naming whilst ignoring visual distractors [three]	All three tasks showed activity in the dACC, in the contrast of difficult trials (incongruent) against rest baseline.	−4,12,36	−	−
	Vocal colour naming while ignoring visual distractors (Stroop task) [Tthree]	In the two language tasks, this area showed increased activity for the difficult (incongruent) relative to easier (congruent) stimuli, consistent with the involvement of domain-general mechanisms of attentional control in word production.			
	Non-language task (Simons task) [two]				

In all these studies activity was related to non-linguistic processing in components of the cingulo-opercular network (Fig. 5B).

Abbreviations are: dACC, dorsal anterior cingulate; aI/IFG, anterior insula/inferior frontal gyrus; dlPFC, dorsolateral prefrontal cortex; R, right; L, left; MNI, Montreal Neurological Institute stereotactic co-ordinates.

We describe two of these studies in more detail. Vaden *et al.* (2013) tested the prediction that elevated cingulo-opercular activity increases the likelihood of immediate correct word recognition. The participants were required to repeat heard words presented against a background of babble noise. The signal-to-noise ratio during speech perception had two levels, so that one repetition task was more difficult and resulted in greater errors. Activity in the cingulo-opercular network increased during the trials with lower signal-to-noise ratio, and activity in this system correlated with error rates across all trials. Further, using partial correlations functional connectivity analyses on each trial, the authors demonstrated that increased cingulo-opercular activity was predictive of better word-recognition performance on the next trial. In a second study, Piai *et al.* (2013) required their subjects to perform three tasks, each with two or three levels of difficulty. Two tasks involved verbal stimuli and responses while the third was a visual task with manual responses (Table 1). Reaction times and errors were longer for the more difficult levels of each task. Activity in the dorsal ACC was increased across all tasks, and in two of the three tasks activity was significantly greater during the more difficult trials.

Unlike the networks described above that activate during ‘exteroceptive’, externally driven tasks, the Default Mode Network (Fig. 3) is an ‘interoceptive’ system with a distinct and reproducible anatomical distribution (Raichle *et al.*, 2001; Esposito *et al.*, 2006; McKiernan *et al.*, 2006; Buckner *et al.*, 2008). It includes midline cortex, located in the ventral anterior and posterior cingulate cortex and the precuneus, but also lateral neocortical regions, the angular gyri (posterior inferior parietal cortices) and rostral dorsolateral prefrontal cortex, and the medial temporal lobes (Fig. 3). Although typically considered a resting state network, part of this network overlaps with those supporting speech production and the semantic processing of verbal stimuli (Seghier *et al.*, 2010; Geranmayeh *et al.*, 2012; Seghier and Price, 2012; Smith *et al.*, 2012). Binder *et al.* (2009) argue that during ‘rest’ human brains are far from inactive, with stimulus-independent thoughts engaging the retrieval of both episodic and semantic memories, and that this function explains activation of components of the Default Mode Network during language processing.

The exteroceptive or task-positive networks demonstrate anticorrelated activity with the interoceptive Default Mode Network (Fox, 2005; Spreng *et al.*, 2010). The balance between the two has been studied in healthy participants (Weissman *et al.*, 2006; Kelly *et al.*, 2008; Leech *et al.*, 2011), and it is now apparent that this balance becomes disrupted during pathological conditions. This has been demonstrated in traumatic brain injury (Bonnelle *et al.*, 2011), Alzheimer’s disease (Zhou *et al.*, 2010), and brain changes during normal ageing (Andrews-Hanna *et al.*, 2007).

These observations are also relevant to stroke. A cerebral hemispheric stroke may be a focal disease of acute onset, but in addition to damage to a limited (albeit often large) region of cortex, it also invariably results in damage to white matter tracts, including long intra- and interhemispheric pathways. It also often includes highly connected subcortical nuclei. The contributions of ‘disconnection syndromes’ to the behavioural consequences of stroke are well recognized (Geschwind, 1965*a*, *b*; Catani, 2005) but those of ageing, microangiopathic cerebrovascular disease as the result of co-existing hypertension and diabetes, and unsuspected neurodegenerative pathology are difficult to assess in individual cases. It is intuitive that these will have an adverse effect on stroke recovery and rehabilitation. This may, in turn, depend on the distributed connectivity between the exteroceptive and interoceptive networks, and hence efficient task-related deactivation of the Default Mode Network, a concept that is entering the literature on the cognitive effects of cerebrovascular disease (Sheorajpanday *et al.*, 2013; Tuladhar *et al.*, 2013).

This role of domain-general networks in recovery from aphasic stroke is becoming recognized in the neuropsychological literature. Although most of the neuropsychological studies on aphasia recovery design their assessments and consider their results in terms of residual function in domain-specific language systems, there is a small literature that has associated communication problems in patients with aphasia with associated impairments of executive control (Robertson and Murre, 1999; Purdy, 2002; Fillingham *et al.*, 2006; Fridriksson *et al.*, 2006; Lambon Ralph *et al.*, 2010; Murray, 2012), attention (Murray, 2000, 2012; Lambon Ralph *et al.*, 2010), problem-solving as measured by Raven’s Coloured Matrices and Wisconsin Card Sorting Test (Gainotti *et al.*, 1986; Baldo *et al.*, 2005), memory (Swinburn *et al.*, 2005; Fillingham *et al.*, 2006; Lambon Ralph *et al.*, 2010; Murray, 2012), semantic control (Corbett *et al.*, 2009), or the ability to inhibit distracting stimuli (Wiener *et al.*, 2004). These non-linguistic verbal cognitive problems seem to have an impact on the response to behavioural therapy (Goldenberg *et al.*, 1994; Robertson and Murre, 1999; Fillingham *et al.*, 2006; Lambon Ralph *et al.*, 2010; Yeung and Law, 2010). Thus, for example, in a study on 33 patients with chronic post-stroke aphasia and naming difficulties (Lambon Ralph *et al.*, 2010), a principle component analysis revealed both a phonological factor and a cognitive factor as best predicting therapy outcome designed to improve the residual anomia.

## Suggested proposals about mechanisms of language recovery after a stroke

Functional neuroimaging studies investigating aphasia recovery usually interpret their results in the context of three broad mechanisms. The first two predate the introduction of functional neuroimaging. The ‘perilesional’ hypothesis proposes that recovery is the consequence of the reconstitution of domain-specific language systems in the tissue around the lesion (Heiss *et al.*, 1999; Warburton *et al.*, 1999; Rosen *et al.*, 2000; Hillis *et al.*, 2006; Winhuisen *et al.*, 2007; Meinzer and Breitenstein, 2008; Szaflarski *et al.*, 2013). The second, the ‘laterality-shift’ hypothesis, is that recovery is attributable to a ‘shift’ of language function to the homotopic cortex in the contralateral hemisphere (Musso *et al.*, 1999; Blasi *et al.*, 2002; Leff *et al.*, 2002; Winhuisen *et al.*, 2005; Saur *et al.*, 2006; Turkeltaub *et al.*, 2012). The third, the ‘disinhibition’ hypothesis, has come out of functional neuroimaging research. It proposes that right-sided activity is the product of loss of transcallosal inhibition. It is further proposed that this contributes little to recovery, and may even hinder it by reciprocal inhibition of any remaining undamaged tissue in the left hemisphere (Belin *et al.*, 1996; Rosen *et al.*, 2000; Blank *et al.*, 2003; Naeser *et al.*, 2004, 2005; Thiel *et al.*, 2006).

A hierarchy for aphasia recovery has been proposed (Heiss and Thiel, 2006) that attempts to incorporate these disparate findings. On their synthesis, the best recovery is achieved by the restoration of the original activation patterns within the network of the dominant hemisphere, which is less likely after large lesions. Compensation may also involve secondary centres of the ipsilateral network, a less efficient reorganization. If most of the ipsilateral perisylvian cortex is infarcted, the least efficient compensation is mediated by homotopic contralesional regions. However, conflicting opinions about the roles of ‘laterality shift’ and ‘transcallosal disinhibition’ result in opposing views about rehabilitative interventions; for example, should one attempt to activate or inhibit a contralateral homotopic region with cortical stimulation techniques to promote recovery?

**Figure 4:**
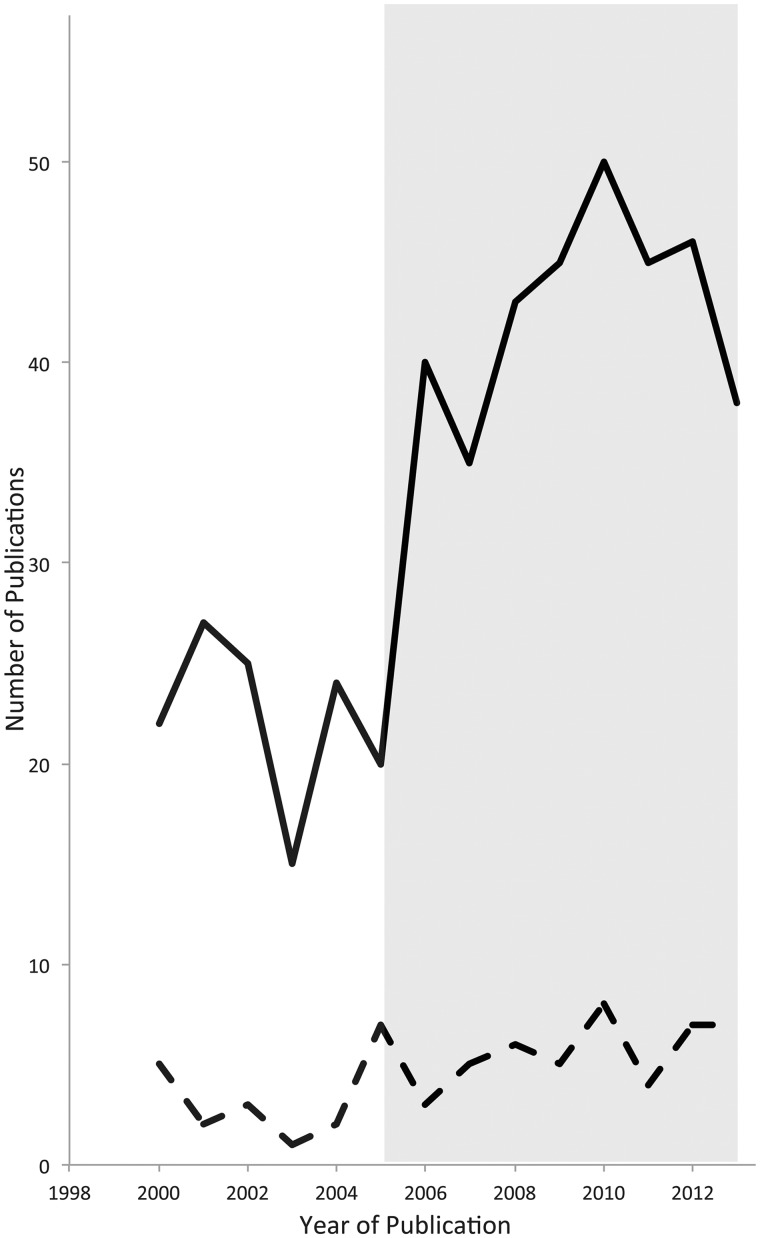
The graph represents the increasing number of publications that have reported functional neuroimaging studies investigating the effects of, or recovery from, cerebral lesions resulting in aphasia. There has been no corresponding increase in interpreting the results from these studies in terms of domain-general cognitive processes. The solid black line represents the annual number of publications returned from the search terms ‘Aphasia AND Functional Neuroimaging’ in PubMed. The dotted line represents the annual number of publications returned from the search terms ‘(Aphasia AND Functional Neuroimaging) AND (Executive OR Cognitive Control OR Conflict OR Attention)’ in PubMed. The shaded area represents the emergence of the parallel literature on domain-general cognitive control networks from functional neuroimaging studies on healthy participants.

Consideration is rarely given in these proposals to the influence of intact domain-general networks on recovery, or the possibility that some of the ‘abnormal’ activity recorded in post-stroke aphasia is the result of the upregulation of normal activity within domain-general networks (Wise, 2003). Thus, activity in response to a language task observed at the margins of a lesion that has affected Broca’s area, or in the homologous region, may not reflect partial domain-specific recovery, but be attributable to activity within intact components of the domain-general cingulo-opercular system. Further, the greatest activity may be observed in the right homologue of Broca’s area in those patients who have shown the least recovery because they have the greatest difficulty with tasks performance, and not because this reflects an ‘inefficient’ domain-specific system for recovery (Heiss and Thiel, 2006), or that it is actively inhibiting recovery (Naeser *et al.*, 2005). Despite an increasing number of published studies that have investigated aphasia using functional neuroimaging in the last decade, the proportion of these studies that correlate recovery with domain-general cognitive processes has remained constant, even though an extensive parallel literature has emerged on these domain-general systems over this period (Fig. 4). In the next sections we will suggest a re-interpretation of specific examples from amongst published studies; but, of course, separating activity in domain-specific from that in domain-general networks is not straightforward without explicit adaptation of study designs, something that has only been performed in a few studies.

## Interpreting task-induced activations in contralateral cortex following left hemisphere lesions

There are earlier studies, using non-neuroimaging techniques and a variety of patient populations that have implicated the right hemisphere in recovery of language-specific functions (Kinsbourne, 1971; Gazzaniga, 1983; Gazzaniga *et al.*, 1984; Papanicolaou *et al.*, 1988; Vargha-Khadem *et al.*, 1997). There have been additional reports of right-handed patients who had recovered some language function after a left hemisphere aphasic stroke, but who then deteriorated further after a second stroke affecting the right hemisphere (Barlow, 1877; Gowers, 1887; Lee *et al.*, 1984; Basso *et al.*, 1989; Cappa and Vallar, 1992; Turkeltaub *et al.*, 2012). However, single-case studies or case series on a few patients are rarely conclusive.

Interpretation of functional neuroimaging studies has not, as yet, allowed a consensus to be reached about the function of the right hemisphere in recovery (Price and Crinion, 2005; Crinion and Leff, 2007). A recent meta-analysis concluded that aphasic patients consistently activated spared left hemisphere language nodes, adjacent left hemisphere cortical regions, and right hemisphere homotopic regions (Turkeltaub *et al.*, 2011). Patients with left inferior frontal lesions recruited right IFG more reliably than those without. It was considered that some regions, including right dorsal pars opercularis, were functionally homologous with corresponding areas in control subjects, whereas others, including right pars triangularis, were functionally dissimilar.

There have been other studies that have used brain stimulation techniques, with or without functional neuroimaging, and have reported a supportive role for the right hemisphere in language recovery after a left hemisphere infarct (Winhuisen *et al.*, 2005, 2007) or gliomas (Thiel *et al.*, 2005). For example, two studies by Winhuisen *et al.* (2005, 2007) applied inhibitory repetitive TMS to both the right and left IFG and measured brain activity using PET in patients with either subacute or chronic post-stroke aphasia. In the subacute setting it was concluded that in about half of the patients the right IFG was ‘essential for language function’. At a later stage after the onset of stroke, the role of the right IFG in supporting single word tasks was demonstrated in a smaller proportion of patients. The study populations were small, and, as in most studies, the language tasks were ‘metalinguistic’, requiring a decision and a response; processes that place demands on attention and executive functions. Therefore, specifically relating activity in residual tissue in the left IFG and in the right IFG to domain-specific language function rather than these other domain-general processes may be too narrow an interpretation of these findings.

The hypothesis that activity in the right IFG may inhibit residual left IFG function in aphasic stroke patients (the ‘disinhibition’ hypothesis) was explicitly investigated in a study based on data from healthy participants (Hartwigsen *et al.*, 2013). A virtual lesion of either the anterior or posterior left IFG with continuous theta burst stimulation was created, and its immediate effects on the repetition of real words and pseudowords assessed. The behavioural effect was very mild, but inhibition of the posterior left IFG resulted in slight slowing of the reaction time during the repetition of pseudowords, but not real words. This was associated with increased activity in the right posterior IFG. Using effective connectivity analysis, the authors showed that the right IFG was influencing activity in the partially inhibited left IFG, and across the group the strength of this connectivity correlated inversely with the slowing of reaction time (‘repetition onset time’). The authors concluded that the right posterior IFG activity was ‘adaptive’ or, to be more precise, showed ‘adaptive plasticity’, the latter term implying a change within a language-specific network. The model used to determine effective connectivity only included two regions, the left and right posterior IFG. Considering the emerging literature on the relationship between these regions and the dorsal ACC/SFG, which together form the cingulo-opercular network, it would have been of interest to observe changes in effective connectivity had the dorsal ACC/SFG been included in their connectivity model. This might have given a very different impression, namely upregulation of top–down control from a domain-general network in response to impaired task performance rather than rapid adaptive plasticity in the right pars opercularis.

One of the most thorough functional MRI study on aphasic stroke patients, cited >300 times, was by Saur *et al.* (2006). We consider that this study is a good exemplar of the difficulties inherent in the interpretation of right IFG activity in recovery. A particular merit of this study is that three functional MRI studies were performed on the majority of their patients: within a few days of the stroke (acute phase), at 2 weeks (subacute phase) and finally at ∼1 year (chronic phase). The lesions were of different sizes and distributions in the left middle cerebral artery territory. Participants performed two tasks. First, a simple task of distinguishing between two heard sentences; normal sentences and unintelligible sentences played in reverse. The patients had to perform significantly better than chance at detecting this gross difference in the stimuli. The second, and more difficult task, was to detect a semantic violation in half the forward sentences (e.g. ‘The pilot flies the plane’ compared with ‘The pilot eats the plane’). Performance on the easier first task earned only a maximum of 10% of the behavioural score for in-scanner performance, whereas performance on the second task could earn up to 90% of the marks (Dorothee Saur, personal communication). Patients improved their performance on both standard tests of language abilities and on within-scanner performance over time. Comparing the raw scores over time, it can be concluded that few patients were able to detect semantic violations in the acute stage. Therefore, it would be reasonable to assume that they attended to the easier task of differentiating between forward and reversed sentences. However, the scores indicate that by 2 weeks post-stroke almost all were ‘having a go’ at the much more difficult semantic violation task, although at the expense of an appreciable number of errors. Then, by 1 year the semantic violation task had become easier, with a performance similar to that achieved by healthy participants. Therefore, the study design included both a change in language scores across the scanning sessions and, plausibly, fluctuating cognitive ‘effort’ in the performance of the more difficult semantic violation task.

**Figure 5:**
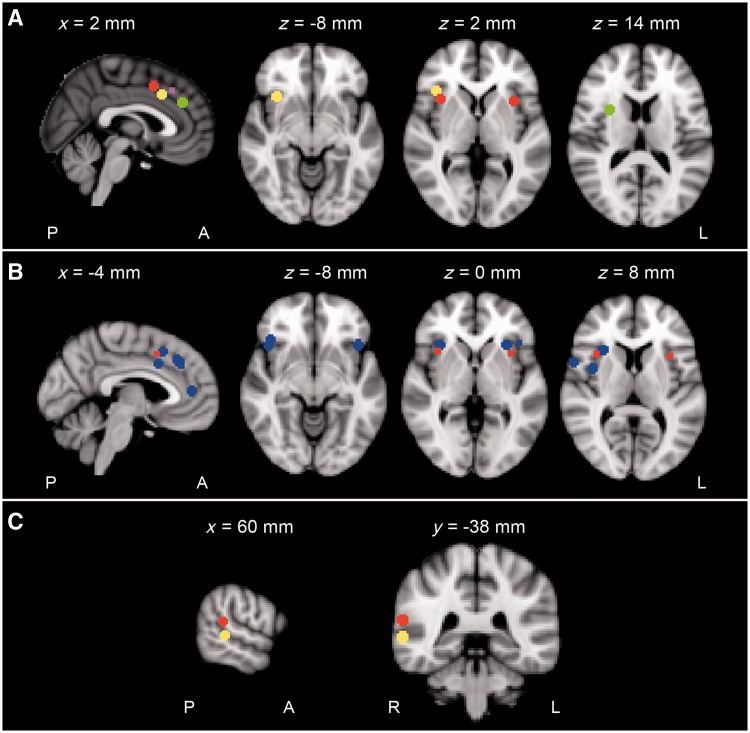
Activation peaks from the neuroimaging studies of language tasks discussed in the text that can be attributed to domain-general systems. Each activation peak is represented as a sphere with a 5 mm radius around the reported peak coordinate of activity, superimposed on a single T_1_-weighted magnetic resonance image, anatomically normalized into the Montreal Neurological Institute standard stereotactic space. (**A**) Activation peaks from studies on patients with stroke that showed a positive correlation with measures of aphasic recovery. These peaks localized to the dorsal anterior cingulate cortex (sagittal view) and right IFG/right anterior insula (axial views). The red regions lie within the ‘cingulo-opercular’ network described by Dosenbach *et al.* (2007). Yellow, purple and green regions are from the studies of Saur *et al.* (2006), Brownsett *et al.* (2014), and Raboyeau *et al.* (2008), respectively. (**B**) Activation peaks from studies on healthy participants that explicitly dissociated language-specific functional MRI activity from that related to domain-general processes. In all these studies activity was related to domain-general processing in components of the cingulo-opercular network. Red region represents peak activity in the ‘cingulo-opercular’ network described by Dosenbach *et al.* (2007). Blue represents activation peaks from studies listed in Table 1. (C) Activation peak (yellow) in the right posterior STS from the study on stroke patients with left posterior temporal infarction by Leff *et al.* (2002). In that study increased activity in the right posterior STS was attributed to a ‘shift’ of language function from the left to the right posterior STS, and was attributed to the recovery of word comprehension. This region is just inferior to the right temproparietal junction that is engaged in attentional processes. Red represents convergent of activity in the right temporoparietal junction related to vigilant attention from a meta-analyis of attentional neuroimaging studies by Langner and Eickhoff (2013). A = anterior; P = posterior; L = left; R = right.

The crux of the interpretation of the results related to what happened to brain activity in the right anterior insular/IFG (Fig. 5A, yellow peaks on axial slices). There was task-related activity at this location in the healthy participants. Activity here was low in the patients at the time of the first scan, but was greater than normal by 2 weeks, before declining to the level observed in the healthy participants by 1 year. This trajectory plausibly follows engagement on the difficult task demand when detecting the semantic violations: little effort at the first time point when the subjects realized the task was too difficult; considerable effort at 2 weeks when there had been partial recovery, resulting in a better performance; and declining activity at 6 months when recovery had made the task much easier. However, the authors dismissed task-related activity as an explanation for their finding, and related it to a dynamic language-specific process contributing to recovery from aphasia after stroke. In contrast, the alternative interpretation was adopted by authors of a study that related right IFG activity in aphasic patients to non-linguistic processing due to task difficulty or learning (van Oers *et al.*, 2010).

It could be argued that inferring participants’ cognitive ‘effort’ across time in the study of Saur *et al.* (2006) is speculative, whereas the language scores provide objective behavioural data. However, the study went further in relating regional activity with an out-of-scanner composite language score achieved by the patients. At the time of the first study, the language score correlated strongly with activity in both the left and right anterior insular/IFG. Further, the improvement in language scores between the acute and second scans correlated with activity in the right anterior insular/IFG and a midline frontal region that, on the coordinates supplied, locates to the dorsal ACC/SFG (Fig. 5A, yellow peaks on the sagittal slice). Although a contribution from language-specific networks to this result is possible, which was the conclusion made by the authors, overall the results would seem to fit as well or better with varying activity in the domain-general cingulo-opercular network; the greater the upregulation of the cingulo-opercular network, the more top–down control was being exerted, and the better the performance on language tasks.

The identification of a correlation between improved language outcome and activation in dorsal ACC/SFG in aphasic patients is in keeping with results published by Raboyeau *et al.* (2008) and a recent study of our own (Brownsett *et al.*, 2014). See Fig. 5A, for peak coordinates of dorsal ACC/SFG in these studies. Both these studies had taken steps to reduce the task performance (i.e. increase task difficulty) in the healthy control group, and were able to relate activity in the dorsal ACC/SFG in aphasic patients and controls to task difficulty and task demands rather than linguistic processing *per se*. In the study by Brownsett *et al.* (2014) the patients were required to listen to a sentence in preparation to repeat that sentence immediately afterwards. They achieved ∼60% accuracy as a group. The healthy control subjects performed the same task, except that in some trials the sentences had been manipulated to reduce spectral information, using an established technique (Sharp *et al.*, 2009; see also a meta-analysis by Adank, 2012). When contrasting listening to the perceptually difficult sentences with the clear sentences in the healthy participants, they clearly demonstrated increased activity in the cingulo-opercular network, which therefore related to task difficulty but not language processing (which must have been greater during perception of the normal sentences). In patients, activity in the dorsal ACC/SFG was observed when they listened and prepared to repeat normal sentences, a task that they found difficult, and this activity correlated with performance on an out-of-scanner overt picture description task. The study was interpreted as demonstrating the influence of domain-general control when task difficulty increased, as the result of perceptual distortion of the stimuli in the healthy participants and aphasic impairment in the patients; and that the individual ability to activate this network influenced outcome after stroke.

In addition to domain-general executive networks, evidence is now emerging from patient studies and neurostimulation of healthy participants, for the existence of controlled access to semantic representations. Contrasting aphasic stroke patients with ‘semantic aphasia’ and patients with the semantic variant of fronto-temporal dementia, indicated that seemingly similar impairments are the result of impaired task-dependent access to semantic representations in the former group and degradation of the representations themselves in the latter group (Jefferies, 2006). The distribution of major pathology in the two groups is quite different, with left fronto-parietal destruction in the stroke patients and bilateral anterior temporal lobe atrophy in the patients with semantic dementia. It is suggested that executive control over semantic processing is dependent on a distributed neural network that includes bilateral dorsolateral prefrontal cortex, the left angular gyrus and left posterior temporal cortex (Thompson-Schill *et al.*, 1997; Wagner *et al.*, 2001; Whitney *et al.*, 2011, 2012; Noonan *et al.*, 2013). This evidence converges with our view on the interpretation of many of the results coming from functional neuroimaging studies.

## ‘Laterality shifts’ in temporo-parietal cortex

Most studies of aphasia recovery that relate recovery to ‘laterality shifts’ of language processing have reported these changes in the inferior frontal cortex. Many fewer studies have reported a similar laterality shift in the other eponymous language region, namely Wernicke’s area in left posterior temporal cortex and adjacent inferior parietal cortex (for examples see Leff *et al.*, 2002; Teki *et al.*, 2013). Although the evidence from lesion studies strongly implicate the left posterior temporal cortex in language processes, a recent functional-anatomical model has proposed that the acoustic analysis of heard speech and lexical access is a function of both the left and right posterior temporal cortex (albeit with a ‘weak left hemisphere bias’) (Hickok and Poeppel, 2007). This model proposes that the language processes that are strongly left-lateralized either decode meaning conveyed by the syntactical structuring and ordering of words to access sentence-level semantics or are central to speech production. On this basis, it might be expected that the homologue of Wernicke’s area can support at least some of the functions associated with the spectrotemporal analysis of heard speech and access to lexical representations. One study in support of this view was that of Leff *et al.* (2002), who demonstrated that the response of the right posterior superior temporal sulcus (STS) changed after chronic aphasic stroke, showing a profile that came to resemble that of the left posterior STS in healthy right-handed subjects. It was proposed that this was due to a reorganization of synaptic function in a domain-specific language. Although this interpretation may be correct, and many consider that the perception as opposed to the production of language is left-lateralized (Hickok and Poeppel, 2007), it is a region just inferior to the right temporoparietal junction, at the intersection of the posterior end of the STS with the inferior parietal lobule and the lateral occipital cortex (Fig. 5C). A meta-analysis of 55 studies has concluded that the right temporo-parietal junction forms part of a distributed network of brain regions mediating vigilant attention (Langner and Eickhoff, 2013). This network consists of top–down and bottom–up attentional processes, and overlaps with other fronto-parietal components of the ventral attentional system (Fig. 3) that respond to salient and behaviourally relevant stimuli (Corbetta and Shulman, 2002; Corbetta 2008). In the model proposed by Langner and Eickhoff (2013), the right temporoparietal junction may be engaged in ‘reorientation signalling’ and become active when attention has drifted away from the task and needs to be refocused. Thus, a response at the posterior end of the right STS that is greater in aphasic patients than in healthy control subjects as they listen to verbal stimuli could plausibly reflect differences in the degree of engagement of the ‘bottom–up’ attentional processes rather than a change in the response of language-specific cortex. This again addresses the issue that aphasic patients and healthy controls differ not only in terms of language processing, but also in the demands on attention and executive control, with aphasic patients almost invariably having to exert increased attention and cognitive control when processing verbal stimuli and perform linguistic tasks.

## Interpreting training induced changes following aphasic stroke

With the exception of the earlier discussion on neurostimulation-induced changes in aphasic stroke, we have so far mainly focused on the brain responses to spontaneous recovery after aphasic stroke. Functional neuroimaging has also been used to investigate training-induced recovery (Musso *et al.*, 1999; Cherney and Small, 2006; Fridriksson *et al.*, 2006; Thompson *et al.*, 2010). However, these studies have included few patients (i.e. <4) (Cherney and Small, 2006; Fridriksson *et al.*, 2006; Meinzer *et al.*, 2007; Vitali *et al.*, 2007) or larger case-series of patients analysed individually (Musso *et al.*, 1999; Meinzer *et al.*, 2008; Thompson *et al.*, 2010), which makes generalization of their findings to the larger population unreliable.

To our knowledge only two studies have looked at training-induced effects using a group level analysis, and both of these have emphasized the role of systems supporting language rather than shift of language function *per se*. Raboyeau *et al.* (2008) studied the effect of lexical training on 10 patients performing a naming task in their native language compared directly with 20 healthy participants completing the same task in a foreign language. They found an increase in activity in the right anterior insular/IFG after training in both groups. The activity in the right anterior insular/IFG correlated with behavioural improvement in patients (see Fig. 5A, green sphere on axial slices) whereas the activity in a right dorsal ACC region correlated with behavioural improvement in both groups. There was also a post-training deactivation in the regions associated with the Default Mode Network, suggesting that all participants were engaging more in the task. The authors interpreted these findings as a neural correlate of lexical learning and suggested that it ‘illustrates the specific monitoring role of the attention network in resolving verbal conflict’. However, the second study (Brownsett *et al.*, 2014), found no neural correlates of training. The authors studied both healthy participants and patients with aphasia while they undertook auditory discrimination training. The authors suggested that this null result may have been due to the use of conventional univariate statistical analyses, which may be too insensitive to reveal the training-induced functional changes. Further neuroimaging studies investigating training induced changes in domain-general brain networks are needed to explore the influence of these networks on training induced recovery after aphasic stroke.

## Practical implications for future study designs

In the absence of valid animal models, the study of recovery of speech and language following aphasic stroke has either to rely on clinical studies or depend on studies on healthy participants and the modulatory effects of task difficulty or the effects of non-invasive brain stimulation. To combat some of the pitfalls in interpreting functional neuroimaging signals in future studies, a few methodological issues need to be considered when designing experiments.

First is the selection of appropriate baseline tasks. Two points need to be taken into account when selecting these tasks: one is an inclusion of an equally demanding ‘non-linguistic’ baseline task. Ideally, this task should match the language task in terms of difficulty as measured by similarities in error rates and reaction times. This is needed in order to help differentiate activations resulting from linguistic networks from domain-general networks. Examples in which this issue was considered include the studies on healthy participants by Eckert *et al.* (2009) and Piai *et al.* (2013) (Table 1). Another issue when interpreting baseline tasks includes the likely modulation of activity in the Default Mode Network. As activity in the Default Mode Network is upregulated during ‘rest’ conditions, many now consider that ‘rest’ or other ‘passive’ conditions have limitations as a baseline condition for subtractive experimental designs that investigate language processing, and incorporate a higher-level baseline task (Spitsyna *et al.*, 2006; Awad *et al.*, 2007).

The second issue relates to the comparative task difficulty of the language task(s) between the aphasic patient group and the healthy control subjects. It has been suggested that this can only be achieved by performing functional neuroimaging studies on patient populations who are able to do a task with comparable error rates and reaction times to the control population (Price and Friston, 1999). This has its obvious limitations, and will exclude a disproportionate number of even relatively mildly affected subjects from a study. As a result, it is a restriction that has clearly been ignored in almost all patient studies. An alternative is to make the same task more difficult for the control participants. Although this may be achieved in speech comprehension paradigms (Sharp *et al.*, 2009; Brownsett *et al.*, 2014), it is more difficult to do in speech production paradigms. Nevertheless attempts have been made to this effect by comparing activations in controls naming objects in a partially learnt foreign language and aphasic patients naming objects (Raboyeau *et al.*, 2008). To emphasize, measuring activity across a range of levels of task difficulty in patients and in control participants may allow the level of difficulty to be matched, and the effect of cognitive control distinguished from language-specific factors.

Third, is the need for longitudinal imaging studies of spontaneous aphasic stroke recovery. To date, only a few studies, on a small number of patients, have performed longitudinal functional neuroimaging studies in aphasic patients from the acute to chronic stage (Heiss *et al.*, 1999; Saur *et al.*, 2006). One limitation of longitudinal studies is the potential for alterations in vascular reactivity throughout the brain acutely after stroke. It may be necessary to measure the vascular reactivity, using, for example, a short breath-hold functional MRI task to observe the response to mild hypercarbia to prove that the lack of activation in stroke patients is not just due to a lack of neurovascular decoupling (Murphy *et al.*, 2011). An application of a similar method was made by van Oers *et al.* (2010), and at least in the chronic phase (>1 year after stroke) this methodological issue seems not to be a confound.

Fourth, multivariate imaging techniques, such as independent component analysis (ICA) (Beckmann and Smith, 2004, 2005), may identify functionally distinct but anatomically overlapping networks that are not always apparent from a subtractive univariate analysis (Leech *et al.*, 2011, 2012; Geranmayeh *et al.*, 2012). ICA takes advantage of low frequency fluctuations in the functional MRI data to separate the signal into spatially distinct components that will include domain-general and domain-specific cognitive networks (Smith *et al.*, 2009). As an example from our own research (Geranmayeh *et al.*, 2012), a simple univariate contrast between participants speaking and generating non-communicative movements of the articulators failed to demonstrate any activity in the left parietal cortex; in fact, if anything, net activity within this region was less than in the baseline condition. However, an analysis using ICA demonstrated that within the left inferior parietal cortex there was a locally distributed subcomponent whose activity correlated strongly with that in both the left IFG (Broca’s area) and left posterior temporal cortex (the other well-known eponymous language region, Wernicke’s area), both regions evident in the univariate analysis.

ICA is emerging as a powerful tool for separating multiple brain networks in healthy controls and patient groups (Filippini *et al.*, 2009; Veer, 2010; Basile *et al.*, 2013; Smith *et al.*, 2013, 2014; Tuladhar *et al.*, 2013). A methodological caveat is that the effects of large lesions, such as stroke, on the validity of multivariate analyses, remains to be investigated.

## Conclusion

This review has argued that the interpretations of functional neuroimaging studies on aphasic stroke recovery often ignore the contribution of upregulated intact domain-general cognitive control systems, and their possible modulation of downstream domain-specific networks. With this in mind, the proposed, and at times conflicting, hypotheses about the mechanisms of language recovery after stroke may need revision, particularly when future studies are designed specifically to assign activity to domain-specific and domain-general networks. This is not just of academic interest. Stroke mostly occurs in subjects over 60 years of age, when age-related cognitive decline or presymptomatic neurodegenerative pathology is becoming established, and longstanding chronic conditions, such as hypertension and diabetes, have affected the microvasculature of the brain. Within this context, an aphasic stroke is acute focal pathology in addition to established diffuse chronic pathology. Many relatively underpowered studies have indicated that age may not affect prognosis after stroke (Plowman *et al.*, 2012), but evidence from a very large stroke registry has concluded that age has a strong influence on outcome (Knoflach *et al.*, 2012). Although the mechanisms underlying this observation are not apparent from this study, one obvious factor is the effect of accumulated impairment of domain-general, distributed brain networks over the lifetime of any individual patient. This interpretation would suggest that rehabilitation should be aimed at improving function in attention and executive function as much as restoring, as far as possible, language-specific processes.

## Abbreviations

ACC: anterior cingulate cortex
IFG: inferior frontal gyrus
SFG: superior frontal gyrus
STS: superior temporal sulcus
